# Acute Intoxication with Caffeine-Containing Tablets: A Case Report with a Fatal Outcome

**DOI:** 10.3390/jox16020056

**Published:** 2026-03-24

**Authors:** Maya Radeva-Ilieva, Stanila Stoeva-Grigorova, Ivanesa Yarabanova, Ivelina Panayotova, Georgi Bonchev, Nadezhda Hvarchanova, Mario Milkov, Simeon Marinov, Petko Marinov, Snezha Zlateva

**Affiliations:** 1 Department of Pharmacology, Toxicology and Pharmacotherapy, Faculty of Pharmacy, Medical University of Varna, 9000 Varna, Bulgaria; stoeva.st@mu-varna.bg (S.S.-G.); nadejda.hvarchanova@mu-varna.bg (N.H.); petko.marinov@mu-varna.bg (P.M.); snezha.zlateva@mu-varna.bg (S.Z.); 2 Clinical Toxicology Department, Naval Hospital, 9000 Varna, Bulgaria; ivanesa_98@abv.bg; 3 Laboratory of Analytical Toxicology, Naval Hospital, 9000 Varna, Bulgaria; stefanova_vma@abv.bg (I.P.); georgi.bontchev@gmail.com (G.B.); 4 Department of Dental Materials Science and Prosthetic Dental Medicine, Faculty of Dental Medicine, Medical University of Varna, 9000 Varna, Bulgaria; mario.milkov@mu-varna.bg; 5 Department of Urology, Faculty of Medicine, Medical University of Varna, 9000 Varna, Bulgaria; dr.marinov.simeon95@gmail.com

**Keywords:** caffeine, acute intoxication, fatal outcome, suicide, HPLC

## Abstract

Caffeine is widely consumed and generally considered safe at customary doses. How-ever, high-dose preparations available online pose a risk of severe and potentially fatal intoxication. Although uncommon, lethal caffeine poisoning is associated with profound cardiovascular and neurological toxicity. A rare case of intentional acute caffeine intoxication with fatal outcome is presented. A 25-year-old woman ingested an estimated 60 tablets containing 200 mg of caffeine each, purchased online. She was admitted to hospital shortly after ingestion of the caffeine tablets with palpitations, agitation, dizziness, and repeated vomiting. On examination, she presented with arterial hypotension (90/60 mmHg) and marked sinus tachycardia (150 beats/min), accompanied by psychomotor agitation. Her blood caffeine concentration measured by means of high-performance liquid chromatography (HPLC) was 177 µg/mL. The patient’s condition rapidly deteriorated, with the development of convulsive syndrome progressing to coma, extreme ventricular tachycardia, exotoxic shock, and toxic cardiomyopathy. Despite intensive care management, including mechanical ventilation and advanced cardiopulmonary resuscitation, the patient died several hours after admission. In conclusion, this case underscores the life-threatening potential of acute high-dose caffeine ingestion and highlights the risk associated with unrestricted access to concentrated caffeine products. Early recognition and aggressive management are crucial, yet may be insufficient in cases of massive overdose.

## 1. Introduction

Caffeine (1,3,7-trimethylxanthine) is the most widely consumed psychoactive substance worldwide and is generally regarded as safe when ingested at customary dietary doses [1]. Its pharmacological activity is primarily mediated through nonselective antagonism of adenosine A_1_ and A_2_A receptors, resulting in disinhibition of neuronal activity and enhanced release of excitatory neurotransmitters, including catecholamines. At higher concentrations, caffeine additionally inhibits phosphodiesterase enzymes and promotes intracellular calcium mobilization, thereby augmenting myocardial excitability and sympathetic stimulation. These mechanisms underlie its central nervous system (CNS) stimulant properties as well as its cardiovascular effects, including positive chronotropic and inotropic responses [2,3]. At moderate doses, caffeine improves alertness, vigilance, and cognitive performance. It has established medical applications, most notably as caffeine citrate in the management of apnea of prematurity, where it reduces apnea frequency and enhances respiratory drive [4,5]. Furthermore, caffeine is widely used as an adjuvant in combination with non-opioid analgesics and non-steroidal anti-inflammatory drugs, potentiating their analgesic efficacy [6].

Daily caffeine intake of up to 400 mg in healthy adults is considered safe by major regulatory authorities, including the European Food Safety Authority (EFSA) and the U.S. Food and Drug Administration (FDA). This intake roughly corresponds to four cups of brewed coffee and is not typically associated with clinically significant adverse reactions [2,7,8]. Nevertheless, even at therapeutic doses, caffeine may cause tachycardia, anxiety, tremor, gastrointestinal discomfort, and sleep disturbances [4]. In contrast, excessive caffeine exposure can result in severe toxicity. Clinical manifestations of acute caffeine poisoning include pronounced agitation, persistent vomiting, seizures, refractory ventricular tachyarrhythmias, and hemodynamic instability. Therapeutic plasma caffeine concentrations typically range between 4 and 8 µg/mL. Symptomatic toxicity is generally observed at plasma concentrations above 15–20 µg/mL, whereas levels exceeding 80–100 µg/mL have been associated with coma and fatal outcomes. Reported fatal ingestions commonly exceed 5–10 g of caffeine, although significant interindividual variability exists [9,10].

Although severe caffeine intoxication remains uncommon, its reported incidence appears to be increasing, likely reflecting the expanding availability of high-dose caffeine products, including concentrated tablets and powdered formulations marketed online. Parallel concerns have emerged regarding energy drink consumption, particularly among children and adolescents. Many commercially available products contain between 80 and 200 mg of caffeine per serving, with some exceeding 300 mg per container. These beverages are frequently consumed rapidly and may contain additional stimulants such as guarana (a supplementary caffeine source), taurine, and other sympathomimetic compounds, potentially amplifying cardiovascular and neuropsychiatric effects [11,12]. A recent systematic review of published case reports spanning more than a century identified over 200 documented cases of acute caffeine toxicity, a substantial proportion involving intentional overdose and severe cardiotoxic or neurotoxic manifestations. While many patients recover with supportive treatment, a subset develops life-threatening arrhythmias, refractory shock, and multiorgan dysfunction, underscoring the importance of early recognition and aggressive management [10].

In this report, we present a rare case of intentional acute caffeine intoxication with fatal outcome in a 25-year-old woman who ingested an unknown quantity of caffeine-containing tablets (estimated 12 g of caffeine according to the available data). This case highlights the life-threatening potential of massive caffeine overdose and emphasizes the public health risks associated with unrestricted access to concentrated caffeine products, especially among young individuals. Early recognition and aggressive management are crucial, yet may be insufficient in cases of massive overdose.

## 2. Detailed Case Description

### 2.1. Medical History

A 25-year-old woman was admitted to the Emergency Consultative Unit of the Military Medical Academy–Varna after a suicide attempt. According to the information obtained from the emergency medical services (EMS) team and partially from the patient herself, approximately one hour prior to hospital admission, the patient intentionally ingested an unknown quantity of caffeine-containing tablets (presumably 60 tablets containing 200 mg of caffeine each, according to the found bottle of tablets) with suicidal intent. It was reported that the tablets had been purchased online. After ingestion, the patient informed her sister about the incident. She experienced multiple episodes of vomiting and was subsequently transported to the hospital by an EMS team. Upon initial examination, the patient complained of palpitations, psychomotor agitation, and dizziness. The patient denied any known medical or psychiatric disorders and reported no regular medication use. However, additional information provided by her parents revealed a previous suicide attempt approximately five years earlier, involving an attempted self-strangulation. Furthermore, two years prior to the current incident, the patient had consulted a psychiatrist for depressive symptoms and had been prescribed homeopathic treatment, which reportedly resulted in clinical improvement, leading to discontinuation of therapy.

### 2.2. Physical Examination and Clinical Course

On admission, the patient was in moderately impaired general condition and was able to maintain an active position in bed. The patient was conscious, communicative, agitated, dysthymic, and oriented, with intermittent episodes of psychomotor agitation. Pupils were equal and miotic, with a mildly delayed light reflex.

**Figure 1:**
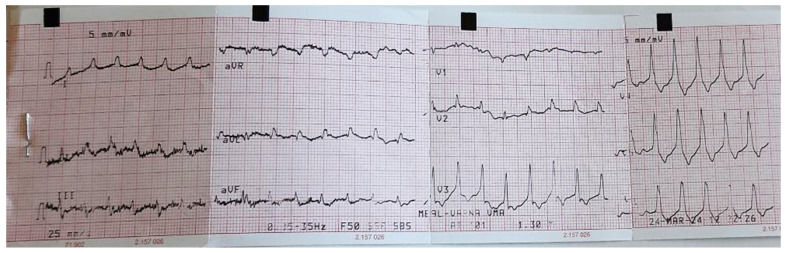
Patient’s ECG result on admission.

Cardiovascular examination demonstrated regular tachycardia rhythm (150 beats per minute), muffled heart sounds, and no pathological murmurs (Figure 1). Blood pressure was 90/60 mmHg.

The exact duration over which the tablets were ingested could not be reliably established; however, based on the available history and clinical presentation, the ingestion is presumed to have occurred over a short period of time, consistent with an acute overdose. The patient’s condition on admission is described in detail in Table 1, which summarizes the patient’s clinical course by hours.

Gastric lavage followed by administration of activated charcoal was performed immediately after hospital admission in an attempt to remove the ingested caffeine. Gastrointestinal decontamination represents a key early intervention in cases of acute caffeine intoxication, particularly when patients present shortly after ingestion. Activated charcoal is widely recommended due to its capacity to adsorb caffeine within the gastrointestinal tract and thereby limit further systemic absorption. Moreover, given that caffeine undergoes enterohepatic recirculation, repeated doses of activated charcoal may further enhance elimination by reducing reabsorption. Gastric lavage may be considered in life-threatening ingestions if performed within a narrow time window after exposure; however, its use remains controversial and must be carefully weighed against the potential risk of complications [9,13,14]. Despite its benefits, in massive overdoses such as the present case, gastrointestinal decontamination alone is often insufficient to prevent severe systemic toxicity due to the rapid absorption of caffeine and the large ingested dose.

### 2.3. Toxicological Screening and Laboratory Results

#### 2.3.1. Urine Drug Screening

Blood and urine samples from the patient were analyzed at the Laboratory of Analytical Toxicology. A 10-panel urine drug screening test was performed (Table 2). The results were negative for all substances tested, except for benzodiazepines, for which a positive result was yielded in the urine. This finding is expected and fully consistent with intravenous diazepam administration after hospital admission. Table 2 presents toxicological findings from the urine sample.

#### 2.3.2. Laboratory Analysis of Blood and Urine Samples

The laboratory findings are indicative of the severity and dynamic progression of the clinical course and are consistent with the underlying pathophysiological mechanisms of caffeine toxicity (Table 3). Significant metabolic and electrolyte disturbances were documented, manifested by severe hyperglycemia, hypokalemia, and lactic acidosis.

The observed marked deviations of some laboratory biomarkers from the listed reference ranges highlight the severity of intoxication.

#### 2.3.3. Toxicological Analysis of Blood Sample

High-performance liquid chromatography (HPLC) analysis was performed using an Agilent 1260 Infinity Binary LC system equipped with a Zorbax Extend-C18 column (150 × 4.6 mm, 5 μm) and a 1260 Infinity diode array detector (DAD) for quantitative determination of caffeine.

The pre-analytical procedure began with 500 μL of plasma sample, alkalinized with 500 μL of 1 M NaOH. Deproteinization was achieved by the addition of 1.5 mL acetonitrile, followed by a two-step liquid–liquid extraction using 2 × 3 mL ethyl acetate. The organic phase was evaporated to dryness, and the residue was reconstituted in 500 μL of mobile phase and filtered through a 0.22 μm nylon syringe filter prior to analysis. Chromatographic separation was carried out under isocratic conditions using a methanol–water mobile phase (25:75, *v*/*v*) at 25 °C with a flow rate of 1.0 mL/min. The injection volume was 20 μL. Detection was performed at a wavelength of 274 nm.

Data acquisition and processing were carried out using the Agilent OpenLAB (ChemStation edition, rev. C.01.05) and MassHunter (rev. B.07.00)software package. All chemicals and reagents were of analytical grade or higher. Mobile phases and standard solutions were prepared using HPLC-grade solvents and purified deionized water (0.067–0.100 μS cm^−1^) obtained from a TKA™ Pacific water purification system.

Under the described chromatographic conditions, the retention time of caffeine was approximately 5.02 min. The measured plasma caffeine concentration was 177 µg/mL. The result of the HPLC analysis was ready on the next day at 10:59 AM and was immediately reported to the Department of Toxicology.

### 2.4. Diagnosis

The patient was diagnosed with severe acute caffeine intoxication (ICD-10 code T43.6) as a result of intentional ingestion of caffeine tablets. The clinical course was complicated by cerebrototoxic syndrome, marked psychomotor agitation, and status epilepticus progressing to coma. The patient developed exotoxic shock, extreme ventricular tachycardia, and toxic cardiomyopathy.

She underwent endotracheal intubation and invasive mechanical ventilation, as well as advanced cardiopulmonary resuscitation. Despite full intensive care support, the clinical course was refractory, and a fatal outcome was recorded approximately six hours after admission to the hospital. No autopsy was performed.

### 2.5. Therapeutic Course

The therapeutic course during the patient’s hospitalization is presented in Table 4 by hours. It reflects a multidisciplinary approach focused on stabilizing the patient’s condition as well as preventing complications and fatal outcome.

## 3. Discussion

The present case illustrates a fulminant course of massive caffeine intoxication characterized by rapidly progressive cardiovascular collapse, refractory ventricular tachyarrhythmia, severe metabolic acidosis, and toxic cardiomyopathy. The measured plasma caffeine concentration of 177 µg/mL, confirmed by means of HPLC analysis, is markedly above levels commonly associated with fatal outcomes (>80–100 µg/mL), providing objective toxicological confirmation of extreme systemic exposure [10,15]. Therefore, the biochemical data corroborate the clinical severity and support the causal relationship between massive caffeine ingestion and the fatal outcome.

Under physiological conditions, caffeine is rapidly and almost completely absorbed from the gastrointestinal tract, with peak plasma concentrations typically occurring within 30–120 min. It is primarily metabolized in the liver by cytochrome P450 1A2 (CYP1A2), with an elimination half-life of approximately 3–7 h in healthy adults. However, in massive overdose, caffeine metabolism may become saturated, resulting in non-linear kinetics and significant prolongation of elimination half-life [3,9,16]. Omi (2021) reported that in severe intoxication, the half-life may extend beyond 15 h due to metabolic pathway saturation [17]. This pharmacokinetic feature may partly explain the rapid escalation of toxicity observed in massive ingestions, as sustained high serum concentrations perpetuate adrenergic stimulation, intracellular calcium overload, and progressive myocardial instability. In the present case, the measured plasma caffeine concentration of 177 µg/mL strongly suggests metabolic saturation, as such levels far exceed the threshold typically associated with severe toxicity.

In the present case, the early clinical presentation of marked sinus tachycardia, hypotension, agitation, vomiting, and subsequent progression to ventricular tachycardia (VT) is consistent with profound sympathetic overstimulation. At toxic concentrations, caffeine exerts competitive antagonism of adenosine A_1_- and A_2_A-receptors, inhibits phosphodiesterase enzymes, and enhances intracellular calcium release through ryanodine receptor activation. The resulting catecholamine surge and calcium overload increase myocardial automaticity and triggered activity, predisposing to malignant ventricular arrhythmias. Thus, malignant arrhythmias may be refractory to standard antiarrhythmic therapy even when administered promptly and appropriately [2,3,9]. Severe hypokalemia likely further amplified arrhythmogenic susceptibility. β_2_-adrenergic stimulation induces intracellular potassium shift, while vomiting and osmotic diuresis contribute to additional losses. Additionally, hypokalemia shortens repolarization and promotes re-entry mechanisms, lowering the threshold for sustained VT [18]. Concurrent severe metabolic acidosis reflects systemic hypoperfusion and β-adrenergic-mediated hyperlactatemia, both recognized features of severe methylxanthine toxicity [9,15,19]. Moreover, at extreme concentrations, caffeine disrupts intracellular calcium homeostasis within cardiomyocytes, impairing excitation–contraction coupling and contributing to acute toxic cardiomyopathy and hemodynamic collapse [2,3]. Elevated intracellular calcium may contribute to triggered activity and complex arrhythmogenic substrates, as evidenced by reported bidirectional VT in heavy caffeine poisoning [20]. The progression from VT to asystole, with only transient return of spontaneous circulation despite advanced cardiopulmonary resuscitation, suggests profound electrical instability rather than primary hypoxic arrest. This pattern has been described in severe caffeine intoxication cases complicated by refractory ventricular arrhythmias and shock [10,21].

Management of severe caffeine intoxication remains primarily supportive and symptom-directed, as there is no universally accepted antidote or standardized treatment protocol due to the rarity of such cases. Initial therapy focuses on stabilization of airway, breathing, and circulation, correction of metabolic derangements such as acidosis and electrolyte imbalances, and control of seizures and arrhythmias with appropriate anticonvulsants and cardiovascular support agents. Beta-adrenergic blockade may be considered for persistent tachyarrhythmias, while correction of hypotension often requires vasoactive support such as dopamine infusion. Continuous monitoring in an intensive care setting is essential given the risk of rapid deterioration [9,22]. Adjunctive measures such as forced diuresis and enhanced elimination have been described in case reports, reflecting attempts to augment toxin clearance; however, the evidence base consists predominantly of individual cases rather than controlled trials [9].

In the management of severe caffeine intoxication complicated by malignant ventricular arrhythmias, antiarrhythmic therapy is foundational, yet its efficacy may be limited by the underlying pathophysiology of methylxanthine toxicity [9,15]. In this case, the patient was treated with intravenous amiodarone and magnesium sulfate, consistent with advanced cardiac life support strategies for refractory ventricular arrhythmias. Amiodarone’s multi-channel blocking effects can suppress ventricular ectopy and reentrant circuits, while magnesium stabilizes cardiomyocyte membranes and may blunt catecholamine-mediated excitability [23,24]. However, case series and toxicology reviews indicate that severe caffeine-induced ventricular arrhythmias can be refractory to standard antiarrhythmic interventions, likely due to persistent adrenergic stimulation and intracellular calcium overload that diminish the effectiveness of pharmacologic suppression. One case report describes persistent ventricular fibrillation following massive caffeine overdose that required prolonged supportive care and ultimately responded only after extracorporeal support modalities were initiated [23]. In our case, guideline-based antiarrhythmic therapy was initiated, including bolus and continuous intravenous amiodarone and intravenous magnesium sulfate to address myocardial electrical instability and catecholamine-mediated arrhythmogenicity [25]. This highlights a mechanistic limitation of conventional antiarrhythmics in the setting of profound methylxanthine toxicity.

Considering the mechanisms of caffeine toxicity, β-adrenergic blockade represents a rational therapeutic approach, as it directly counteracts catecholamine-mediated cardiotoxicity. Several toxicology reviews support the use of β-blockers in cases of severe caffeine intoxication complicated by refractory tachyarrhythmias [9,26,27]. In the present case, bisoprolol was administered. Despite aggressive antiarrhythmic management and the administration of magnesium sulfate and bisoprolol, the arrhythmia proved refractory and was accompanied by progressive hemodynamic instability requiring cardiopulmonary resuscitation and mechanical ventilation.

In addition to cardiovascular stabilization and antiarrhythmic therapy, the patient received comprehensive supportive treatment, including benzodiazepine administration, electrolyte correction, vitamin supplementation, and neuroprotective measures. The use of diazepam is consistent with current toxicology recommendations for the management of agitation and seizure activity in caffeine intoxication, as benzodiazepines are considered first-line agents for methylxanthine-induced CNS hyperexcitability. Correction of electrolyte disturbances is particularly important in severe caffeine poisoning, as hypokalemia and metabolic derangements contribute significantly to arrhythmogenesis and myocardial instability [9]. Supportive adjuncts such as B-group vitamins and neuroprotective agents are frequently employed in critical care settings to mitigate secondary metabolic and neurologic injury, particularly in patients with convulsive syndromes and evolving cerebral toxicity. Although direct evidence for outcome modification in caffeine poisoning is limited, their use aligns with established principles of neuroprotective supportive care in toxic encephalopathy [28,29].

Extracorporeal elimination techniques, particularly hemodialysis and hemoperfusion, have been reported in severe or life-threatening caffeine overdoses and may be effective in reducing serum concentrations. Caffeine possesses several physicochemical properties that make it amenable to extracorporeal removal, such as low molecular weight (194 Da), relatively low protein binding (≈15–35%), small volume of distribution (~0.6 L/kg), and water solubility [30]. In published case series, hemodialysis has been associated with clinical improvement and reduced toxin levels, although timing and indications remain case-specific rather than guideline-driven [31,32]. One such report demonstrates venoarterial extracorporeal membrane oxygenation (ECMO) support in the context of caffeine-induced ventricular fibrillation with subsequent favorable outcome [33]. In the present case, extracorporeal elimination was not pursued. The rapid clinical deterioration with early onset of asystole likely precluded the time necessary for vascular access and initiation of dialysis or ECMO. This underscores a major practical limitation in caffeine poisoning management, as even theoretically effective treatments may not be implementable.

Intravenous lipid emulsion (ILE) has been proposed as an adjunctive therapy in cases of severe drug intoxication, particularly for highly lipophilic agents, based on the “lipid sink” theory. Although caffeine is only moderately lipophilic and exhibits relatively low protein binding, isolated case reports have described the use of ILE in massive caffeine poisoning complicated by refractory cardiovascular instability [34]. Kohl et al. (2020) discuss the potential role of lipid emulsion therapy in non-local anesthetic toxicities, noting that evidence outside classical lipophilic cardiotoxins remains limited and largely anecdotal [35]. Similarly, Muraro et al. (2016) emphasize that lipid emulsion therapy should be considered a rescue intervention when conventional resuscitative measures fail, particularly in life-threatening cardiotoxicity [36]. Controlled clinical data, however, remain lacking. In the present case, ILE was also administered to the patient.

An overall comparison with previously reported cases of severe caffeine intoxication reveals several consistent clinical patterns. Most fatal or near-fatal cases involve ingestion of large quantities of caffeine in the form of tablets or powder, frequently exceeding 5–10 g, and are characterized by rapid onset of tachyarrhythmias, central nervous system excitation, and metabolic derangements. Ventricular tachycardia and ventricular fibrillation are among the most commonly reported life-threatening complications [9,10,15]. A systematic review by Uehlein et al. (2025) identified more than 200 published cases of acute caffeine toxicity, with a significant proportion presenting with malignant arrhythmias and hemodynamic instability. Importantly, cases with plasma caffeine concentrations exceeding 100 µg/mL were strongly associated with severe outcomes, including cardiac arrest and death [10].

Despite these common features, the available literature demonstrates considerable heterogeneity in both clinical course and therapeutic response, reflecting differences in ingested dose, timing of intervention, and availability of advanced supportive measures. Similar to our patient, several case reports describe refractory ventricular arrhythmias despite aggressive antiarrhythmic therapy [21,34,35,36,37,38,39]. Unlike our case, Bioh et al. (2013) performed continuous venovenous hemodiafiltration, which, according to the authors, contributed to the patient’s survival [38]. Additionally, Elbokl et al. (2021) reported the use of prolonged hemodialysis for caffeine elimination, facilitating patient recovery [30]. Moreover, Harsten et al. (2020) demonstrated that the combined use of intravenous lipid emulsion and hemodialysis may represent an effective strategy in life-threatening caffeine poisoning [34]. However, Han et al. (2022) as well as Pina Cabral et al. (2022) reported successful outcomes with supportive care alone, without the use of active elimination methods such as hemodialysis or ECMO [21,39].

This variability highlights a critical gap in the current evidence base, as treatment decisions are largely guided by case reports rather than standardized protocols. In particular, the timing of extracorporeal elimination appears to be a key determinant of outcome, with favorable results more often reported when such interventions are initiated before the onset of profound hemodynamic collapse. In contrast, rapidly progressive toxicity, as observed in the present case, may preclude the timely implementation of these potentially life-saving measures.

Although rare, fatal outcomes following caffeine ingestion have been documented in association with a variety of formulations, including tablets, powders, energy drinks, and concentrated caffeine products. Intentional overdoses involving caffeine tablets or bulk powder are the most commonly reported causes of lethal intoxication, often due to the ease of access to high-dose preparations via online markets [40]. Case series and individual reports indicate that fatal intoxication typically presents with sudden cardiac arrest, seizures, and malignant arrhythmias, frequently occurring shortly after ingestion of large quantities of caffeine. In a series of fatal cases, postmortem blood concentrations commonly exceeded 80 mg/L, with deaths primarily attributed to arrhythmogenic mechanisms [41,42,43,44]. More recent reports confirm that fatal outcomes continue to occur, including a case involving combined caffeine and methamphetamine intoxication, highlighting that although rare, caffeine-related deaths remain consistently documented in the literature [45].

Overall, the available evidence indicates that while moderate caffeine consumption is generally safe, high-dose exposure carries a substantial risk of life-threatening toxicity and sudden cardiac death.

In conclusion, the present case illustrates the fulminant clinical course that may follow massive caffeine ingestion and emphasize that therapeutic success may be limited by rapid toxic progression and refractory cardiovascular instability. The case further highlights the importance of early consideration of extracorporeal removal techniques in patients with markedly elevated serum concentrations of caffeine, provided that clinical conditions permit timely initiation.

Finally, an additional noteworthy aspect in this case is the patient’s documented history of depressive symptoms and a prior suicide attempt, for which psychiatric consultation had been sought. According to available information, treatment consisted solely of homeopathic preparations. It is important to emphasize that current evidence does not support the clinical effectiveness of homeopathy in the management of depression [46]. Untreated or inadequately treated depression is a well-established risk factor for suicidal behavior, and failure to implement evidence-based psychiatric management may increase vulnerability to future suicide attempts. In this context, reliance on non-evidence-based therapeutic approaches may represent a missed opportunity for effective intervention and long-term risk reduction.

## 4. Conclusions

Acute massive caffeine ingestion can result in rapidly progressive, life-threatening cardiotoxicity and neurotoxicity, even when prompt and aggressive supportive treatment is provided. The present case underscores the potential for fatal outcome at extremely elevated plasma concentrations and highlights the limitations of current therapeutic options in fulminant intoxication. Early recognition, intensive monitoring, and timely consideration of extracorporeal elimination strategies are essential, although they may not always be feasible in rapidly deteriorating patients. Furthermore, the unrestricted availability of high-dose caffeine products, including energy drinks, represents an emerging public health concern and underscores the need for greater awareness and regulatory oversight.

## Figures and Tables

**Table 1:** Clinical timeline.

Hour	Clinical Course
11:45 AM	⋅ The patient complained of palpitations, psychomotor agitation, and dizziness. ⋅ The patient was in moderately impaired general condition. She was afebrile and had a normosthenic body habitus. The skin appeared pale, while the visible mucous membranes were pink. ⋅ The patient was conscious, communicative, agitated, dysthymic, and oriented, with intermittent episodes of psychomotor agitation characterized by carpopedal spasms (“obstetrician’s hand” posture). She denied hallucinations or other psychotic symptoms. Pupils were equal and miotic, with a mildly delayed light reflex. Assessment for nystagmus could not be adequately performed due to poor cooperation. There was no characteristic odor on the breath and no visible traumatic injuries to the head or body. Traces of emesis were noted around the mouth. ⋅ Respiratory examination revealed a respiratory rate of 18 breaths per minute, with vesicular breath sounds and no wheezes or crackles bilaterally. The patient’s oxygen saturation was 96% on room air. ⋅ Cardiovascular examination demonstrated sinus tachycardia with a heart rate of 150 beats per minute, muffled heart sounds, and no pathological murmurs. Blood pressure was 90/60 mmHg. ⋅ Abdominal examination showed a soft, non-tender abdomen with normal bowel sounds. The liver and spleen were not enlarged. Costovertebral angle tenderness was absent bilaterally. Examination of the extremities revealed no abnormalities. ⋅ Therapeutic interventions: ⋅ Gastric lavage was performed with activated charcoal; ⋅ A urethral catheter was placed, yielding 100 mL of clear urine; ⋅ Continuous cardiovascular monitoring was initiated; ⋅ Physical/mechanical restraint was applied; ⋅ A urine toxicology screening was performed (10-panel urine drug screen test).
02:00 PM	⋅ The patient remained in a severely impaired general condition; ⋅ She was responsive to verbal stimuli but restless, with ineffective retching and spastically increased muscle tone; ⋅ Respiratory rate was 18 breaths per minute, with vesicular breath sounds and no wheezes or crackles bilaterally. Oxygen saturation was 96% on room air; ⋅ Cardiovascular examination revealed muffled heart sounds and persistent hypotension (85/60 mmHg); ⋅ The abdomen was soft and non-tender; ⋅ Examination of the extremities showed no abnormalities; ⋅ Diuresis was stimulated with an intravenous administration of 20 mg of furosemide (10 mg/mL, 2 mL) and 150 mL of a 10% mannitol solution, resulting in a urine output of 100 mL; ⋅ A consultation with an anesthesiologist–intensivist was appointed.
02:40 PM	⋅ The patient was poorly responsive and disoriented. ⋅ The skin and visible mucous membranes were cyanotic, and violaceous mottling was observed on the lower extremities. ⋅ Ophthalmologic examination revealed divergent strabismus. Pupils were constricted with delayed light reflex. Respiratory rate was 10 breaths per minute, with clear bilateral vesicular breath sounds and no adventitious sounds. ⋅ Hemodynamically, the patient exhibited extreme tachycardia (heart rate 220 beats/min) and unmeasurable blood pressure. Urine output was 100 mL of clear urine. ⋅ Following consultation with an anesthesiologist–intensivist, the patient was transferred to the intensive care unit (ICU) for further management.
02:45 PM	⋅ The patient was transferred to the ICU in a state of toxic shock. She was unresponsive and clinically unstable. ⋅ The skin and visible mucous membranes were cyanotic. Multiple violaceous skin discolorations were observed over the body, predominantly on the lower extremities. ⋅ Respiratory examination revealed bilaterally present, rapid vesicular breath sounds without added adventitious sounds. Oxygen saturation was 82%. ⋅ Hemodynamically, she was severely compromised, with extreme tachycardia (heart rate 220 beats/min) and non-measurable arterial blood pressure. ⋅ Therapeutic interventions: ⋅ The patient was endotracheally intubated and placed on invasive mechanical ventilation with 100% oxygen. Oxygen saturation subsequently increased to 98%; ⋅ A central venous catheter was inserted into the superior vena cava via the right internal jugular vein, and infusion of intravenous solutions was initiated. Central venous pressure was measured at 24 cm H_2_O; ⋅ A nasogastric tube was placed, yielding a small amount of black gastric content.
02:47 PM	⋅ The patient was evaluated by a cardiologist. ⋅ The patient was in toxic shock. She was afebrile. ⋅ Blood pressure was 50/30 mmHg, with a heart rate of 200 beats/min. Electrocardiographic monitoring demonstrated ventricular tachycardia. ⋅ Respiratory examination revealed vesicular breath sounds without added wheezes or crackles. ⋅ The extremities were free of edema.
04:45 PM	⋅ The patient developed asystole, and full advanced cardiopulmonary resuscitation was initiated.
05:08 PM	⋅ Following resuscitation, return of spontaneous circulation was achieved, with a heart rate of 135 beats/min and blood pressure of 69/50 mmHg.
05:30 PM	⋅ Recurrent asystole occurred, and full advanced cardiopulmonary resuscitation was initiated.
06:15 PM	⋅ Despite cardiopulmonary resuscitation, the patient died.

**Table 2:** Results from the 10-panel urine drug screening test (Hangzhou Alltest Biotech Co., Ltd., Hangzhou, Zhejiang Province, China).

Substance	Cut-Off (Urine, ng/mL)	Result
Amphetamine	1000	−
Benzoylecgonine (Cocaine)	300	−
Δ9-Tetrahydrocannabinol metabolite (Marijuana)	50	
Benzodiazepines	300	+
Tricyclic Antidepressants	1000	−
Barbiturates	300	−
Opioids (Morphine, Heroin, Codeine)	300	−
Methadone	300	−
Methamphetamine	1000	−
3,4-Methylenedioxymethamphetamine (MDMA; Ecstasy)	500	−

**Table 3:** Monitoring of Laboratory Parameters.

Parameter	Reference Range	12:09 PM	12:13 PM	03:50 PM	03:57 PM
**ESR [mm/h]**	<20			10	
**Hemoglobin [g/L]**	120–160	145		**116**	
**Hematocrit [L/L]**	0.35–0.55	0.447		0.362	
**Erythrocytes [×10^12^/L]**	3.6–5.4	4.77		3.79	
**Leukocytes [×10^9^/L]**	3.5–10.5	**18.18**		**24.44**	
**Neutrophils [×10^9^/L]**	2.4–6.9	**10.03**		**15.76**	
**Lymphocytes [×10^9^/L]**	0.8–3.4	**6.87**		**7.54**	
**Monocytes [×10^9^/L]**	0.4–1.0	0.95		0.8	
**Eosinophils [×10^9^/L]**	<0.3	0.26		0.26	
**Basophils [×10^9^/L]**	<0.1	0.07		0.08	
**Immature Granulocytes [×10^9^/L]**	<0.3	0.15		**1.32**	
**Platelets [×10^9^/L]**	140–440	401		327	
**Lymphocytes [%]**	22–50	37.8		30.9	
**Neutrophils [%]**	37–80	55.2		64.4	
**Eosinophils [%]**	1.5–8	1.4		**1.1**	
**Basophils [%]**	<1	0.4		0.3	
**Immature Granulocytes [%]**	<4	0.8		**5.4**	
**Monocytes [%]**	2–10	5.2		3.3	
**MCV [fL]**	82–100	93.7		95.5	
**MCH [pg]**	28–32	30.4		30.6	
**MCHC [g/L]**	300–360	324		320	
**RDW [%]**	11.5–14.9	12.2		12.1	
**MPV [fL]**	8.8–12.5	9.9		10.1	
**NRBC [×10^9^/L]**	<0.01	0		0	
**NRBC [%]**	<0.01	0		0	
**CRP [mg/L]**	<5		3.3		
**Blood Glucose [mmol/L]**	3.88–5.83		**17.9**	**30.4**	
**α-Amylase [U/L]**	29–103		**229**		
**Albumin [g/L]**	35–53			37	
**Total Protein [g/L]**	66–83			**55**	
**Urea [mmol/L]**	2.8–7.2		5		
**Creatinine [μmol/L]**	53–106		93	118	
**AST [U/L]**	<32		25		
**ALT [U/L]**	<34		21		
**GGT [U/L]**	<32		8		
**Total Bilirubin [μmol/L]**	5–21		8		
**Bilirubin (urine dipstick)**	<4				Negative
**Urobilinogen (urine dipstick)**	<17				Normal
**pH (urine dipstick)**	5.0–8.0				≤5
**Specific Gravity**	1.001–1.030				≥1.030
**Protein (urine dipstick)**	Negative				**Positive**
**Glucose (urine dipstick)**	Negative				**Positive**
**Ketones (urine dipstick)**	Negative				**Positive**
**Nitrites (urine dipstick)**	Negative				Negative
**Leukocytes (urine dipstick)**	0–5				Negative
**Blood in urine**	Negative				**Positive**
**Sodium [mmol/L]**	135–150			**133**	
**Potassium [mmol/L]**	3.5–5.5			**2.7**	
**Fibrinogen F-1 [g/L]**	2–4			**1.73**	
**BE (ecf) [mmol/L]**	±2.5	**−10.0**		**−18.3**	
**BE (b) [mmol/L]**	±2.5	**−9.8**		**−17.3**	
**HCO_3_ act [mmol/L]**	21–25	**18.0**		**11.1**	
**HCO_3_ stat [mmol/L]**	21–25	**16.9**		**11.7**	
**O_2_ Sat [%] (venous)**	60–80	**52.7**		**99.4**	
**pCO_2_ [kPa]**	4.67–6.0	**6.3**		4.8	
**pH**	7.35–7.45	**7.2**		**7.11**	
**pO_2_ [kPa]**	10–13.3	**4.5**		**35.8**	
**tCO_2_ [mmol/L]**	25–33	**19.2**		**12.0**	
**Lactate [mmol/L]**	0.5–2.2	**10.3793**		**10.0674**	

Bold values indicate clinically significant abnormalities. Red values indicate elevated level. Blue values indicate decreased level.

**Table 4:** Patient’s therapeutic course and interventions by hours.

Hour	Treatment	Dose and Route	Therapeutic Goal
**11:45 AM**	Sodium chloride 0.9%	500 mL i.v., 2×/day	IV hydration and electrolyte balance restoration
	Glucose 5%	500 mL i.v., 2×/day	IV hydration and electrolyte balance restoration
	Ringer	500 mL i.v., 1×/day	IV hydration and electrolyte balance restoration
	Famotidine	20 mg i.v., 2×/day	Reduction in stomach acidity
	Citicoline	500 mg i.v., 1×/day	Neuroprotective and vitamin therapy
	Thiamine	80 mg i.v., 3×/day	Neuroprotective and vitamin therapy
	Pyridoxine	100 mg i.v., 3×/day	Neuroprotective and vitamin therapy
	Magnesium sulphate	1 amp/4095 mg i.v., 1×/day	Electrolyte correction; Management and prevention of arrhythmias; Adjuvant for enhancing hemodynamic stability, reducing muscle contractility and CNS excitation
	Diazepam	10 mg i.m. in the emergency department; 10 mg by i.v. infusion, and as needed	Anxiolytic/psychotropic therapy
	Haloperidol	5 mg i.m., and as needed	Psychotropic therapy
	Bisoprolol	5 mg p.o., and as needed	Heart rate control
	Oxygen	3 L/min via face mask	Oxygen therapy
	Furosemide	20 mg i.v. as needed	Diuretic therapy and renal support
	Dexamethasone	8 mg i.v., and as needed	Anti-inflammatory therapy; Prevention of cerebral edema
	Mannitol 10%	150 mL i.v.	Diuretic therapy; Prevention of cerebral edema
**02:00 PM**	Furosemide	20 mg i.v. as needed	Diuretic and renal support
	Human albumin 20%	100 mL i.v.	Restoration and maintenance of blood volume
	Gelofusine 4%	500 mL i.v.	Restoration and maintenance of blood volume
	Intravenous lipid emulsion 20%	500 mL i.v., infused over 3 h, as needed	Parenteral nutrition; Lipid rescue therapy
	Dopamine	Continuous infusion via perfusor	Hemodynamic and renal support
	Citicoline	500 mg i.v.	Neuroprotective and vitamin therapy
	Thiamine	80 mg i.v., 3×/day	Neuroprotective and vitamin therapy
	Pyridoxine	100 mg i.v., 3×/day	Neuroprotective and vitamin therapy
	Anticonvulsant	as needed	Anticonvulsant therapy
**02:45 PM**	Propofol	100 mg i.v.	Sedation in the ICU for mechanically ventilated patients
	Endotracheal intubation and initiating mechanical ventilation	100% O_2_	Maintenance of adequate gas exchange
	Nasogastric tube insertion		Enteral feeding and nutrition
**02:47 PM**	Amiodarone	300 mg i.v. bolus 150 mg i.v., infused over 30 min 300 mg i.v., infused over 3 h 300 mg i.v., infused over 6 h	Antiarrhythmic therapy
	Magnesium sulphate	2 amp/8190 mg i.v.	Electrolyte correction; Management of arrhythmias; Adjuvant for enhancing hemodynamic stability, reducing muscle contractility and CNS excitation
	Noradrenaline	1 amp. i.v., continuous infusion via perfusor	Vasopressor, to restore blood pressure
	Sodium bicarbonate 8.4%	3 amp. i.v.	Correction of metabolic acidosis
	Potassium chloride 14.9%	3 amp. i.v., infused with Sodium chloride solution 0.9%, 500 mL	Electrolyte correction
**04:45 PM**	Cardiopulmonary resuscitation		Maintenance of blood flow and oxygenation
**05:30 PM**	Cardiopulmonary resuscitation		Maintenance of blood flow and oxygenation

## Data Availability

The original contributions presented in this study are included in the article. Further inquiries can be directed to the corresponding author.

## Abbreviations

The following abbreviations are used in this manuscript:

HPLC	High-Performance Liquid Chromatography
CNS	Central Nervous System
EFSA	European Food Safety Authority
FDA	Food and Drug Administration
EMS	Emergency Medical Services
ICU	Intensive Care Unit
VT	Ventricular Tachycardia
ECMO	Extracorporeal Membrane Oxygenation
ILE	Intravenous Lipid Emulsion
